# Performance Comparison of Multi-Modal Fusion Techniques in Tissue Perfusion Analysis Using Homography Calibration

**DOI:** 10.3390/s26144585

**Published:** 2026-07-20

**Authors:** Kerim Kursat Cevik, Brendan Tran Morris, Barry Claman, John Menezes

**Affiliations:** 1 Department of Management Information Systems, Akdeniz University, Antalya 07070, Türkiye; 2 Department of Electrical Engineering, University of Nevada, Las Vegas, NV 89154, USA; brendan.morris@unlv.edu; 3 Department of Plastic and Reconstructive Surgery, University of Nevada, Las Vegas, NV 89154, USA; barryclaman@gmail.com (B.C.); john.menezes@unlv.edu (J.M.)

**Keywords:** calibration, deep learning, fusion techniques, tissue perfusion

## Abstract

This study explores the impact of fusion techniques and camera calibration on tissue perfusion analysis using multi-modal imaging. Building on the existing TTPD dataset collected with a prototype hardware platform, we focus on optimizing data alignment and fusion strategies to improve classification accuracy. The imaging system integrates infrared (IR), thermal, and RGB cameras, capturing complementary information about tissue perfusion. To improve modality alignment, we apply Homography-based calibration, reducing spatial discrepancies between different imaging sources. Furthermore, we evaluated early and late fusion approaches using deep learning models (ResNet50 and ResNet101) to determine the most effective integration strategy. Experimental results demonstrate that late fusion, particularly the combination of thermal and RGB modalities, achieves the highest classification performance and that Homography-based alignment improves results. These findings highlight the importance of precise calibration and modality selection in the development of robust and non-invasive tissue perfusion monitoring systems.

## 1. Introduction

Tissue perfusion plays a crucial role in various physiological and pathological processes, making its monitoring essential for clinical applications [1]. An accurate and real-time assessment of perfusion is critical in fields such as surgery, critical care, and vascular disease diagnostics [2]. Continuous and reliable monitoring of tissue perfusion is especially paramount in complex reconstructive procedures, such as vascularized composite allotransplantations and free flap transfers, where the early detection of vascular compromise dictates graft survival [3]. Traditional methods for evaluating tissue perfusion rely on invasive techniques, which may introduce risks and complications [4], or require significant training and attention from care providers. As a result, non-invasive imaging-based approaches have gained significant attention.

Multi-modal imaging, which integrates information from different sensor modalities such as Laser Speckle Contrast Imaging (LSCI) and thermal and RGB cameras, offers a more comprehensive analysis of tissue perfusion [5]. Each modality provides unique insights: near-IR LSCI captures blood flow dynamics, far-IR thermal imaging detects temperature variations, and RGB imaging offers structural details [6] and discoloration associated with occlusion. However, individual modalities have inherent limitations, making fusion-based approaches more effective in capturing a holistic view of tissue perfusion [7].

Deep learning models, particularly convolutional neural networks (CNNs), have demonstrated remarkable success in medical imaging tasks, including segmentation, classification, and anomaly detection [8]. Fusion techniques, such as early fusion, channel-based fusion (2-4-5 channels), and late fusion, aim to combine multi-modal data to improve model robustness and accuracy [9]. Despite recent advances, challenges remain to ensure accurate alignment between different modalities, handle cross-modal variations, and optimize fusion strategies for clinical applications [10]. Furthermore, the literature from the last five years highlights the growing trend of utilizing advanced deep learning architectures, such as spatial–temporal fusion frameworks, to better handle multi-modal medical imaging challenges [11,12]. Studies have shown that even relatively low-resolution thermal sensors can effectively detect perforators [13,14,15,16,17,18]. Several non-invasive and invasive technologies have been developed to improve the accuracy of clinical examinations [19], but none have been universally adopted.

While the various sensing modalities are meant to target differing physiological responses to occlusion, prior work did nothing to ensure calibration between the modal images. This results in suboptimal performance due to differing camera resolutions and viewing angles causing misalignment. The CNN is forced to make more complicated connections at higher layers (larger receptive fields for camera view offsets). With proper alignment, the network can learn multi-spectral features at the lowest level, which are expected to improve context and classification performance.

In this study, we evaluated the effectiveness of different fusion techniques in tissue perfusion analysis using ResNet50 and ResNet101 models. The study is conducted on the Tourniquet Tissue Perfusion Dataset (TTPD) which is divided into two experiments: Pass2 (calibrated dataset) and Unseen (generalization dataset). Additionally, we introduced Homography-based calibration to enhance the spatial alignment of multi-modal images, aiming to improve fusion performance.

The experimental results provide insight into the impact of fusion strategies and calibration methods on deep learning-based perfusion analysis. They highlight the value of image alignment and the need for larger tissue perfusion datasets.

## 2. Hardware and Data Collection

Our work extends the tissue perfusion classification work of Soto et al. [20] by focusing on the camera calibration that was previously missing. We briefly highlight the dataset and explain our calibration process.

### 2.1. TTPD

The Tourniquet Tissue Perfusion Dataset (TTPD) was collected at the University of Nevada, Las Vegas, with a multi-modal data acquisition prototype device. Three different methods were used to obtain the relevant images for each volunteer. The system consists of a thermal IR camera (FLIR A700, Teledyne FLIR, Wilsonville, OR, USA, 640×480 resolution), an RGB camera (Arducam, Nanjing, China, 1200×1200 resolution), and a Laser Speckle Contrast Imaging (LSCI) modality obtained from a customized Arducam camera with the IR filter removed (2000×2000 resolution). Temporal synchronization among the three modalities was achieved via a software-based trigger system, ensuring that frames were captured simultaneously with a minimal millisecond delay tolerance, which is highly sufficient given the 1 frame-per-second (fps) recording rate.

The participants had images taken each second while experiencing arterial and venous blockage by applying a tourniquet to simulate various cases of tissue perfusion. The first 60 s pre-pressure phase (0pre) was used for baseline measurements, while the next 120 s phase caused arterial blockage (1tq1), and the next 60 s phase resulted in venous occlusion (2tq2).

Data was collected with 10 college-aged volunteers (mean age: 21 years) and was divided into two separate experimental scenarios. In Pass2, training was performed on all 10 individuals and testing was conducted on three individuals who were imaged a second time. Crucially, the Pass2 scenario evaluates session-based generalization (i.e., identical subjects recorded during a separate session). With the Unseen set, training was performed on seven individuals and testing was conducted on the three remaining unseen participants, which represents strict subject-based generalization. Table 1 details the exact distribution of frames utilized across all splits.

**Figure 1:**
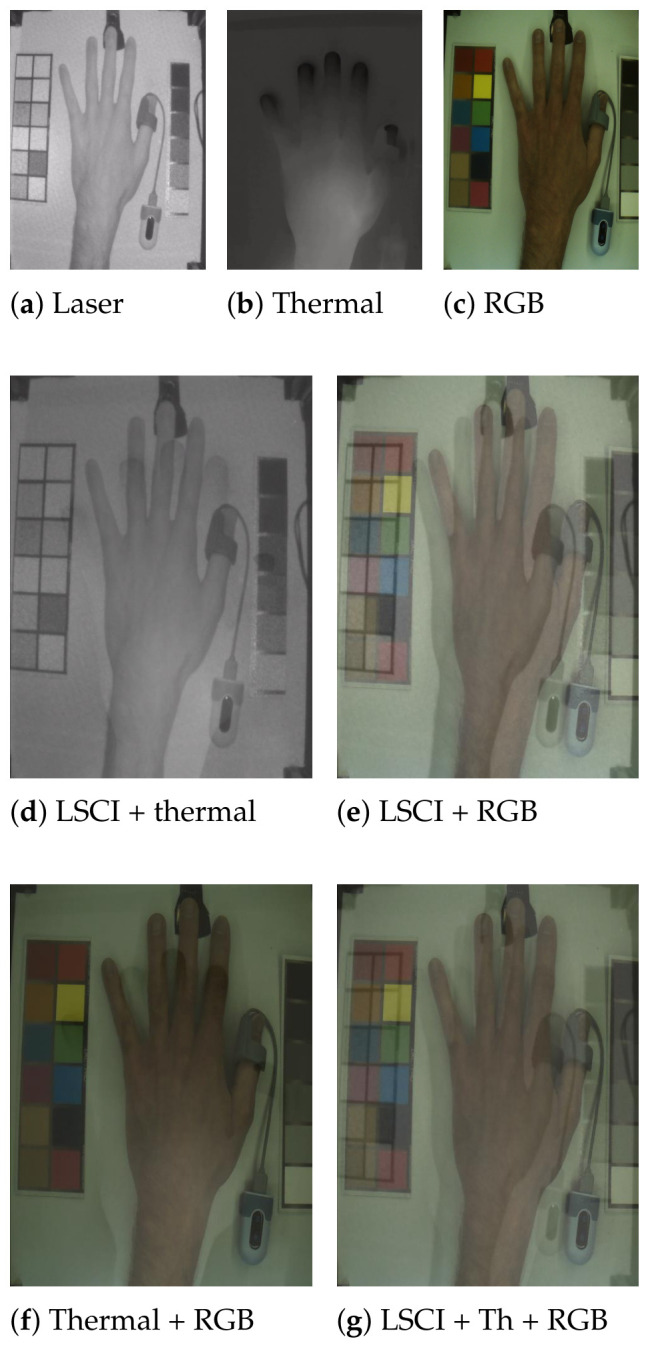
Sample image overlays without calibration.

**Figure 2:**
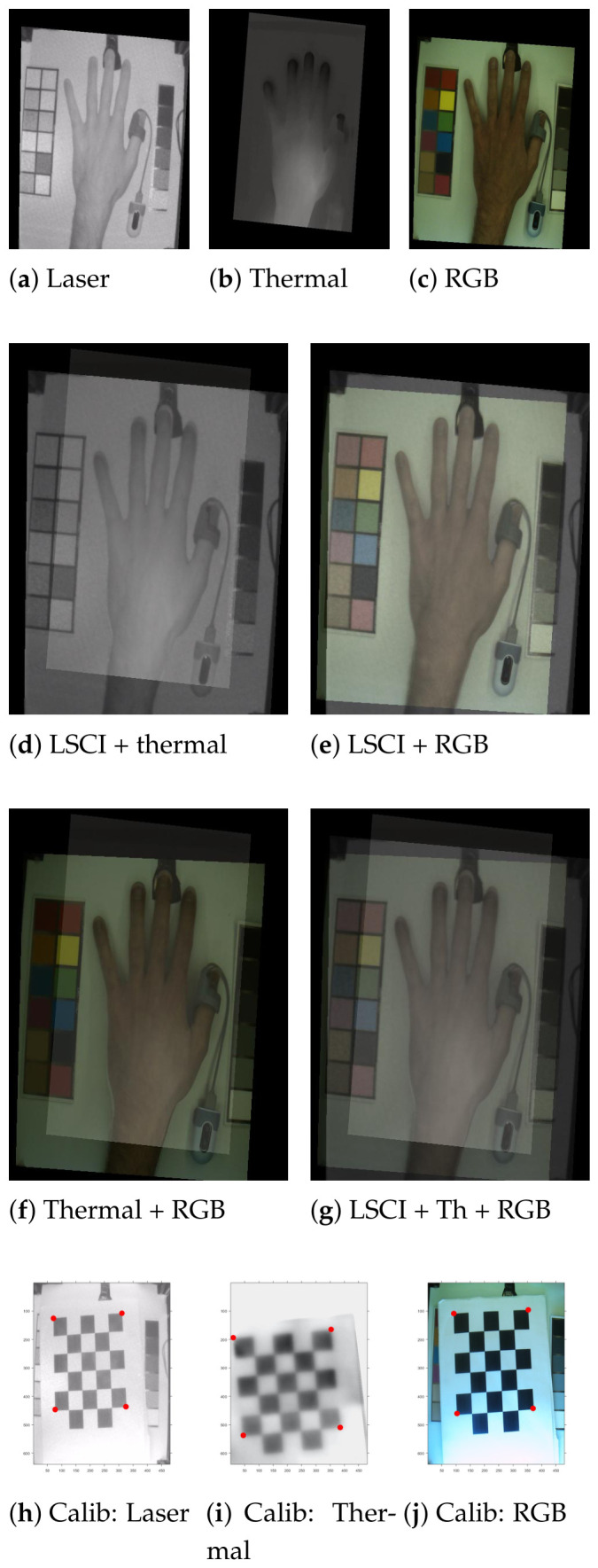
Sample image overlays with calibration (**a**–**g**). The bottom row (**h**–**j**) shows the checkerboard images used for Homography ground plane calibration.

Samples of the uncalibrated and calibrated data are presented in Figure 1 and Figure 2. It should be noted that this pilot dataset consisted primarily of fair-skinned individuals, with only three of the ten participants being female.

### 2.2. Calibration

Prior work directly used resized images for classification and showed some promising results. However, this resizing process resulted in aspect ratio distortion when matching the size of the laser and RGB images to the low-resolution thermal image. Additionally, without calibration, early fusion stacks unaligned images which may diminish the feature extraction power of a CNN. In this work, we explore if appropriate calibration can provide improved performance.

A standard checkerboard calibration approach was utilized to image the experimental volume (bottom of Figure 2). The laser and RGB cameras provide crisp images; however, the thermal images were poor. The checkerboard pattern was left in the sun so that the black squares would heat up more than the white and become visible. The heat quickly dissipated resulting in blurry edges and corners. Therefore, full multi-camera 3D calibration was not possible. Stereo calibration between laser+RGB would work but no stereo was possible with thermal images as corners could not be reliably located.

Instead, a Homography-based calibration method [21] was used to align the ground plane of the experimental volume. The four corresponding corner points on the checkerboard were manually selected for each modality to compute the transformation matrix against a standardized rectangular grid. While formal point-perturbation stability analysis was not conducted, careful manual visual verification and the subsequent improvements in downstream classification accuracy empirically validate the robustness of the estimated matrices. The Four Point Algorithm uses the corner points of the checkerboard (red dots at the bottom of Figure 2) to produce a Homography transformation capable of warping all images into a common ground-plane view. Following the Homography warp, images were adjusted using bicubic interpolation. Because the transformation aligns the modalities to a physically uniform checkerboard grid, aspect ratio distortion is inherently corrected. The out-of-bounds boundary areas resulting from the perspective warp were uniformly zero-padded. As CNNs leverage spatial translation invariance, these zero-padded regions act as a consistent static background across all calibrated images, preventing them from being learned as artificial discriminative features.

Figure 1 shows example images along with overlays without any calibration. There is a clear offset between images when overlayed. The LSCI+RGB example in particular shows two O2 monitors and the hand has a large ∼60 pixel offset. In contrast, Figure 2 shows the same images after Homography transformation. There is strong alignment between the hands across the different modalities, which is expected to provide better features for classification. It should be noted that, due to the Homography transformation, images that are warped may have blank areas (black regions at the top) and exhibit only partial overlap along the edges. Additionally, the Homography transformation assumes a flat ground plane and therefore, anything above the ground (i.e., the hand thickness) will exhibit some distortion.

## 3. Fusion and Classification

### 3.1. Classifiers

In this study, we utilized ResNet50 and ResNet101 due to their proven effectiveness in feature extraction and deep representation learning for medical image analysis. ResNet50, with its 50-layer deep architecture, provides a balanced trade-off between computational efficiency and performance, making it suitable for handling complex patterns in angiographic images while maintaining feasible training times [22]. On the other hand, ResNet101, a deeper variant with 101 layers, allows for the capture of more intricate hierarchical features, which can be beneficial for distinguishing subtle differences in vascular structures [23]. During preliminary experiments, lightweight CNN architectures (e.g., SqueezeNet, GoogleNet) were also evaluated but exhibited lower classification accuracy on this complex multi-modal dataset. Furthermore, while vision transformers (ViTs) and medical transformer encoders offer massive capacity, they are notoriously data-hungry and highly prone to severe overfitting on constrained datasets due to their lack of inductive biases. Therefore, ResNet50 and ResNet101 were intentionally selected as the primary backbone networks to establish a robust, well-understood, and optimal baseline that naturally resists overfitting on limited medical data. Utilizing these standardized CNN backbones allows this study to strictly isolate and quantify the specific performance gains contributed by the Homography-based spatial calibration and the multi-modal fusion strategies, mitigating the confounding effects of architectural complexities. The selection of these models enables a comparative evaluation of network depth’s impact on detection performance while ensuring robust feature extraction capabilities.

### 3.2. Fusion

**Figure 3:**
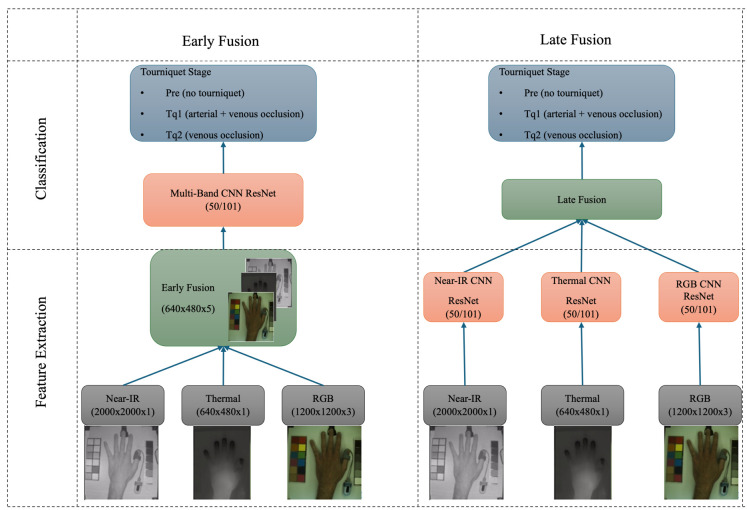
Proposed multi-modal fusion architecture for tissue perfusion analysis. The workflow illustrates early and late fusion strategies specifically targeting the near-IR, thermal, and RGB sensing modalities integrated with ResNet backbones.

The multi-modal nature of the TTPD allows for the detection of different cues: blackening/discoloration with RGB imaging, temperature drop with thermal imaging, and surface vessels with laser imaging. Feature fusion (Figure 3) provides a mechanism to combine the various sensors in a single network and leverage their complementary nature. It is important to note that highly complex, state-of-the-art fusion algorithms could potentially obscure the fundamental improvements gained purely from spatial alignment. By employing basic, well-understood early and late fusion methods, we ensured that the observed performance increases are directly attributable to the Homography calibration.

For feature fusion, some of the different feature fusion methods (early and late fusion) proposed in the literature [24,25] have been applied for perfusion analysis [20]. Early fusion (left-hand side of Figure 3) combines image modalities as separate channels before modeling (CNN network). Late fusion (right-hand side of Figure 3) treats each modality separately, each with an optimized modeling network, and combines the individual results to obtain the final output [20].

Early fusion was applied in two different ways: early blend and 2-4-5 channel. Early blend is designed for direct use of the ResNet architectures without any modification. To ensure a standardized three-channel input ($I_{EB}$), we apply an arithmetic mean across the *N* selected modalities ($I_{1},I_{2},\ldots ,I_{N}$):

(1) $$I_{EB}=\frac{1}{N}\sum_{i=1}^{N}I_{i}$$

where *N* represents the number of modalities being fused (e.g., N=2 for thermal + RGB, N=3 for LSCI + thermal + RGB). This generalized formulation treats all input modalities with equal weighting, ensuring that each sensor contributes proportionally to the fused feature map without bias.

With early 2-4-5 channel fusion, image modalities are combined through channel stacking. Laser and thermal images are treated as a single grayscale channel, while RGB is treated as three channels. The final fusion image $I_{245}$ is built by channel-wise concatenation cat(.):

(2) $$I_{245}=cat\left(cat\left(I_{1},I_{2}\right),I_{3}\right)$$

To accommodate the four- and five-channel inputs within the ResNet architecture, the pre-trained ImageNet 3-channel weights of the first convolutional layer were averaged and replicated across the additional input channels, allowing the network to leverage learned feature extractors seamlessly.

## 4. Results

In this work, we evaluated three individual modalities, four early blend combinations, four early 2-4-5 channel stackings, and four late fusion variants, resulting in a total of 15 unique classifier configurations per backbone for each of the two test scenarios (Pass2 and Unseen) to determine the best way to distinguish our three occlusion situations.

### 4.1. Implementation Details

All model variants are trained using ImageNet [26] pre-trained weights. The hyperparameters used across all tests are as follows: Optimizer: ‘sgdm’; Momentum: 0.9; $L_{2}$ Regularization (Weight Decay): $1\times 10^{-4}$; Learning Rate: $1\times 10^{-4}$ (kept constant); Max Epochs: 10; and MiniBatchSize: 16. Early stopping was monitored on the validation set parameters to prevent overfitting before reaching the maximum epoch limit.The computer used for the tests is equipped with an AMD Ryzen 3 2200G processor with Radeon Vega Graphics (3.50 GHz), 32 GB of RAM, an NVIDIA GeForce RTX 3060 graphics card with 12 GB of VRAM, and a 120 GB SSD with read and write speeds of 500 MB/s. In addition, the computer runs on the Windows 11 operating system and the tests were coded and executed using MATLAB 2024a software (MathWorks, Natick, MA, USA). On average, each test was completed in approximately 1.5 h. Crucially, dataset partitioning for the Unseen generalization test was strictly executed at a patient level. In contrast, the Pass2 scenario evaluates session-based generalization using repeat scans from the same participants. Distinguishing these scenarios ensures that we can evaluate both longitudinal session consistency and strict cross-subject generalization without data leakage.

### 4.2. Calibration Comparison

The full classification comparison results are presented in Table 2 and give an in-depth comparison of multi-modal fusion techniques for tissue perfusion analysis. Results for the two test scenarios are provided: Pass2 ()repeated individuals) and Unseen ()out-of-training-testing) with and without Homography calibration and with different backbone architectures.

Among individual modalities, RGB imaging consistently outperformed laser and thermal imaging, with the highest accuracy of any individual modality except for ResNet101 with Homography for which thermal imaging performed slightly better. Thermal imaging exhibited high performance in the Pass2 dataset but the performance dropped significantly in the Unseen dataset, indicating reduced generalization. LSCI alone performed the weakest, suggesting that it benefits most from fusion with other modalities. The three-channel early blend fusion (LSCI + thermal + RGB) achieved the highest performance before calibration, with an accuracy of 79.5% in the Pass2 dataset using ResNet50. However, its performance dropped to 60.26% in the Unseen dataset, highlighting challenges in generalization. The 2-4-5 channel early fusion approach showed mixed results, with LSCI + RGB achieving 73.45% accuracy in Pass2, but its generalization to the Unseen dataset was less consistent. Late fusion performed competitively, particularly in the thermal + RGB combination, which outperformed all other fusion methods with an accuracy of 87.96% (Pass2, ResNet50) before calibration. The full late fusion (LSCI + thermal + RGB) method performed well with 80.14% (Pass2, ResNet50) but slightly decreased for the Unseen dataset. Among fusion methods, late fusion (thermal + RGB) achieved the best post-calibration results, reaching 86.69% (Pass2, ResNet50) and 75.78% (Unseen, ResNet50) and confirming the advantage of multi-modal alignment.

Homography calibration significantly improved performance, especially for single-modality and late fusion methods. The calibrated RGB-only model improved from 75.49% to 82.54% (Pass2, ResNet50) and from 61.71% to 68.17% (Unseen, ResNet50). Table 3 provides a summary of average performance boost with Homography calibration. Homography generally improved performance, with maximum gains in the Pass2 experiments of 7.65% for ResNet50 Early 2-4-5 and 4.03% for ResNet101early blend. The Unseen test saw a −3.56% decrease for ResNet50 Early 2-4-5 but much higher 11.63% increase for Early 2-4-5.

As expected, there is a large drop in performance going from trained individuals to fully generalizing to Unseen. The smaller performance drop observed with ResNet101 compared to ResNet50 may potentially indicate that its higher capacity is better utilized for generalization when properly calibrating alignment, although it is acknowledged that Pass2 and Unseen evaluate fundamentally different generalization paradigms (session vs. subject). Ultimately, the poor generalization for the Unseen test highlights the need for larger datasets.

### 4.3. Timing Analysis

The TTPD was designed with balanced but random selection of images for training, validation, and testing purposes as is traditional in computer vision. However, the data has strong temporal correlations which could be exploited. In particular, it takes time after the tourniquet pressure is changed before the occlusion causes a change in the hand. During this transition period, accurate classification is more difficult.

**Figure 4:**
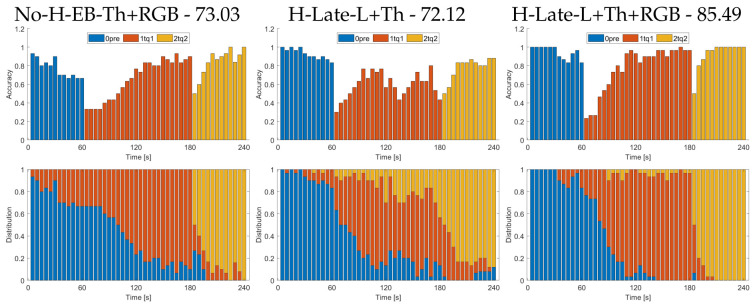
Timing analysis. **Top:** classification accuracy. **Bottom:** classification distribution. The accuracy plots show an increasing trend as the perfusion phase progresses, followed by a drop right after the tourniquet pressure change.

The classification results as a function of time during the tourniquet test are shown in Figure 4 below. Regardless of calibration, the classifiers all struggle after a change in tourniquet pressure phase, as expected. In the top row, there is a clear trend of increasing classification accuracy as the phase progresses in both 1tq1 (orange) and 2tq2 (yellow). In contrast, the initial 0pre phase has a general trend of decreasing performance, which may be related to confusion caused by training on images from the 0pre-1tq1 transition.

The bottom row shows the classification distribution as a function of time. The best performing classifier (H-Late-L+Th+RGB) is able to effectively separate the different phases, with the majority of samples correctly classified. However, the other two show more confusion between classes. There is a large number of 1tq1 (orange) predictions in the early t<60 s 0pre phase for No-H-EB-Th+RGB and 2tq2 (yellow) across the 60≤t<180 s 1tq1 phase. In all three cases, there are too many 0pre predictions across the 1tq1 and 2tq2 times.

In Table 4 we show the classification accuracy accounting for transition delay between 0pre-1tq and 1tq-2tq. This explicit analysis was conducted to quantify and mitigate the labeling noise caused by the inherent physiological lag. By systematically filtering out the non-stationary transition window where the physiological ground truth is ambiguous, the impact of labeling noise is minimized. The lag indicates the number of seconds after the transition that are ignored when calculating accuracy separately by phase. Since there is no transition for 0pre, the accuracy during this phase is constant. By ignoring more time during the transition period, the classification is improved for all three models presented. Practically, this means that the system should be able to detect occlusion after some delay. Additionally, there may be an upper bound on performance as it may not be possible to distinguish the first second into 1tq1 because there is no physical change yet.

Currently, the 1tq phase (arterial inflow occlusion) is the most difficult to correctly distinguish with the lowest accuracy at all lags for all three classifiers.

### 4.4. Feature Space Analysis

**Figure 5:**
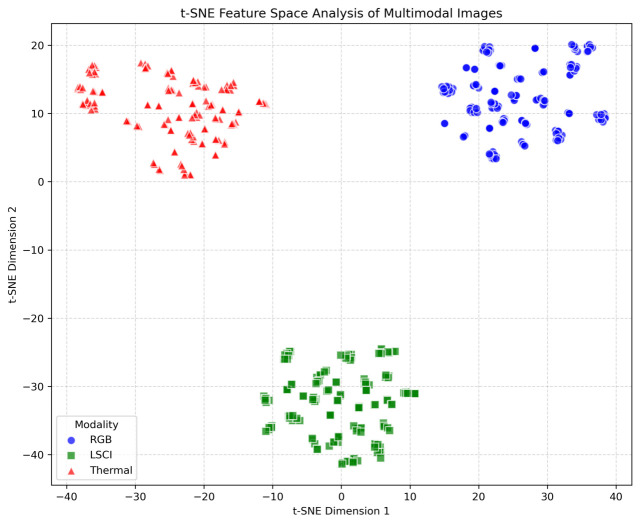
t-SNE feature space analysis of multi-modal images. The distinct clustering of RGB, thermal, and LSCI modalities demonstrates that each sensor captures complementary physiological information.

To empirically substantiate the claim that the three modalities capture complementary perfusion cues, we performed a deep feature space analysis. Deep feature vectors were extracted from the penultimate layer of the pre-trained ResNet50 for a balanced subset of spatially calibrated RGB, thermal, and LSCI images. Subsequently, t-SNE dimensionality reduction was applied. As shown in Figure 5, the deep features from RGB, thermal, and LSCI form distinct, easily separable clusters. This mathematical distinction confirms that even when the cameras are perfectly aligned (via Homography) to view the exact same tissue area, each sensor extracts unique, non-redundant, and complementary physiological information (e.g., surface color, temperature, and blood flow velocity).

## 5. Discussion

A key methodological consideration in our multi-modal early fusion framework concerns the resolution alignment across the distinct imaging spectrums. While the near-IR (2000 × 2000) and RGB (1200 × 1200) data captured high-frequency structural and surface capillary details, they were downsampled to match the spatial dimensions of the thermal sensor (640 × 480) to construct the joint 640 × 480 × 5 tensor. Although this spatial scaling inevitably reduces fine-grained localized texture resolution, the early fusion strategy demonstrated robust classification capabilities by successfully capturing cross-spectral feature correlations from the initial layers of the deep network. This implies that the macro-level spatial distribution of thermal per-perfusion patterns, when coupled with multi-spectral tissue characteristics, provides highly discriminative representations for classifying tourniquet stages, effectively mitigating the impact of localized resolution loss. Future iterations of this work will explore multi-scale feature pyramid networks to leverage native resolutions simultaneously without encountering architectural misalignment.

In this study, spatial registration across the multi-modal sensors was achieved by leveraging a planar Homography calibration matrix. It is important to acknowledge that the hand is a three-dimensional deformable object, and Homography fundamentally assumes a planar geometry, which inevitably introduces minor parallax registration errors at the curved extremities. Furthermore, traditional full 3D stereo calibration relies on checkerboard targets which suffer from rapid heat dissipation, making precise 3D corner detection with thermal sensors highly unreliable. Due to this hardware constraint, and because state-of-the-art (SOTA) 3D medical image registration pipelines incur significant computational overhead prohibitive for real-time monitoring, 2D Homography was implemented. This purposeful trade-off prioritizes low-latency, real-time inference while maintaining robust spatial alignment for the macroscopic regions of interest.

Given the substantial parameter footprint associated with deep convolutional architectures like ResNet-101, mitigating the risk of overfitting on a clinically constrained dataset was a critical priority in our experimental design. To safeguard against this, we heavily utilized transfer learning protocols with pre-trained weights, restricting fine-tuning primarily to the newly introduced multi-modal fusion layers. Furthermore, tissue perfusion and blood flow dynamics are inherently non-stationary processes, which poses a challenge for static artificial neural networks (ANNs). Our transition lag analysis (Table 4) effectively mitigated the labeling noise caused by this physiological delay by excluding the ambiguous transition window. Future studies will explore spatiotemporal networks, such as LSTMs or temporal vision transformers, to better model these dynamic, non-stationary transitions.

Additionally, while this pilot study primarily reports overall accuracy to validate the technical feasibility of the multi-modal calibration, we acknowledge that accuracy alone may mask class-specific performance. Due to the constrained 10-subject sample size, rigorous paired statistical testing or bootstrap confidence intervals were not conducted. Future large-scale clinical evaluations using our upgraded V2 hardware will encompass comprehensive class-wise metrics (e.g., F1-score, sensitivity, specificity, and precision) and rigorous statistical significance testing to firmly establish the superiority of specific fusion strategies across diverse clinical conditions.

## 6. Conclusions

This work extends the tourniquet tissue perfusion study of Soto et al. [20] to consider calibration for alignment of multi-modal images. Similar to the prior study, the use of multi-modal cues is beneficial for identifying arterial and venous occlusion in the hand. In particular, the combination of thermal IR and RGB images had the best performance and late fusion performed better than early fusion approaches. Further, this work shows that Homography calibration can be used to align the images, which provides a performance boost for classification. Deeper analysis of the classification highlighted that most of the errors occurred in the small time window after the tourniquet pressure is changed. It is likely due to the delay before the tourniquet-induced occlusion manifests any physical signs, which suggests an upper limit to classification performance on the TTPD. Finally, we acknowledge that the current dataset serves as a proof-of-concept pilot study, and its limited demographic diversity (10 predominantly light-skinned subjects) is a constraint for immediate clinical generalizability. To directly address this, our ongoing consecutive research is focused on developing an upgraded, high-resolution hardware prototype and actively collecting a larger-scale, demographically diverse multi-modal dataset. Future work will leverage this forthcoming expanded dataset to explore sequence-based accumulation of evidence, spatiotemporal modeling, and advanced architectures (e.g., vision transformers) to further solidify the broader clinical applicability of the proposed tissue perfusion monitoring system.

## Figures and Tables

**Table 1:** Frame distribution across training, validation, and testing sets.

Split	Total Frames	0pre	1tq1	2tq2
**Pass2 (Train)**	4737	1199	2399	1139
**Pass2 (Val)**	450	150	150	150
**Pass2 (Test)**	1420	360	720	340
**Unseen (Train)**	4778	1199	2399	1180
**Unseen (Val)**	450	150	150	150
**Unseen (Test)**	1379	360	720	299

**Table 2:** Perfusion classifier accuracy. Accuracies are presented as percentages.

		Pass2				Unseen			
		No-Homography		Homography		No-Homography		Homography	
Fusion	Modality	ResNet50	ResNet101	ResNet50	ResNet101	ResNet50	ResNet101	ResNet50	ResNet101
Only One	Laser	42.39	41.69	50.28	43.45	44.53	45.90	37.30	49.67
	Thermal	70.28	67.89	69.79	75.63	47.43	46.63	52.50	59.97
	RGB	75.49	77.46	82.54	74.86	61.71	54.02	68.17	68.09
	**Average**	**62.72**	**62.35**	**67.54**	**64.65**	**51.22**	**48.85**	**52.66**	**59.24**
Early blend 3 channels	Laser + RGB	61.62	69.72	78.66	75.92	48.88	53.01	63.24	55.98
	Laser + thermal	41.62	42.82	36.69	44.22	40.25	41.33	45.83	50.91
	Thermal + RGB	73.03	73.38	67.46	73.94	63.52	59.17	73.46	71.50
	Laser + thermal + RGB	79.50	78.87	71.08	86.83	60.26	66.57	54.53	47.57
	**Average**	**63.94**	**66.20**	**64.30**	**70.23**	**53.23**	**55.02**	**59.27**	**56.49**
Early 2-4-5 channels	Laser + RGB	69.44	73.45	80.00	80.28	53.44	44.45	57.58	60.26
	Laser + thermal	36.13	34.23	46.76	43.59	40.03	31.11	36.84	47.43
	Thermal + RGB	70.00	73.87	70.63	68.45	59.32	62.00	58.84	70.20
	Laser + thermal + RGB	69.01	65.07	77.82	69.01	56.56	53.01	41.84	59.17
	**Average**	**61.15**	**61.66**	**68.80**	**65.33**	**52.34**	**47.64**	**48.78**	**59.27**
Late	Laser + RGB	65.49	71.83	78.80	72.89	54.14	57.94	57.94	58.01
	Laser + thermal	67.25	64.79	72.12	69.93	54.68	54.17	50.83	64.03
	Thermal + RGB	87.96	75.28	86.69	79.23	63.38	54.68	75.78	76.36
	Laser + thermal + RGB	80.14	80.21	85.49	76.90	58.67	56.13	65.63	66.57
	**Average**	**75.21**	**73.03**	**80.78**	**74.74**	**57.72**	**55.73**	**62.55**	**66.24**

**Table 3:** Summarized H calibration contributions.

Modality	Pass2		Unseen	
	ResNet50	ResNet101	ResNet50	ResNet101
Only One	+4.82	+2.30	+1.44	+10.39
Early blend 3 channels	+0.36	+4.03	+6.04	+1.47
Early 2-4-5 channels	+7.65	+3.67	−3.56	+11.63
Late	+5.57	+1.71	+4.83	+10.51

**Table 4:** Performance with transition lag ignored.

Model	Phase	Lag [s]					
		0	5	10	15	20	25
No-H-EB-Th+RGB	0pre	77.22	77.22	77.22	77.22	77.22	77.22
	1tq	66.11	67.54	69.09	70.79	72.67	74.39
	2tq	83.24	86.45	89.29	91.20	92.27	92.11
	all	73.03	74.41	75.69	76.77	77.71	78.30
H-Late-L+Th	0pre	92.78	92.78	92.78	92.78	92.78	92.78
	1tq	59.31	60.58	61.52	62.38	63.00	63.33
	2tq	77.35	80.00	82.50	84.00	84.09	84.21
	all	72.11	73.53	74.69	75.56	76.02	76.34
H-Late-L+Th+RGB	0pre	94.44	94.44	94.44	94.44	94.44	94.44
	1tq	77.92	80.29	82.73	85.40	87.33	89.12
	2tq	92.06	96.13	97.86	99.20	99.55	100.0
	all	85.49	87.65	89.23	90.81	91.78	92.68

## Data Availability

The data that support the findings of this study are available from the corresponding author upon reasonable request.
